# The Book World of Medicine and Science

**Published:** 1901-10-12

**Authors:** 


### The Book World of Medicine and Science.

Physical Diagnosis of Diseases of the Chest. By Richard C. Cabot, M.D. (London: Baillière, Tindall and Cox. 1901. Price 10s. 6d. net.)

We have carefully read through this book and we feel sure that it will be found very useful not only by students, for whose use it is primarily intended, but also by many practitioners whose education in physical diagnosis dates from some time ago. In the preface the author says with a certain *naïveté*, "This book is intended for students and, so far as I am aware, contains nothing original." But he adds that he has written it because he has not been able to find any small book upon the subject which does not contain glaring errors, the correct books being too large, and the small books being out of date. At any rate the increase of knowledge during recent years has been so great that we shall none of us be the worse for reviewing what is really to many of us the very foundation of our knowledge of diseases of the chest, namely, the whole subject of physical diagnosis. After a short introductory chapter on regional anatomy we come to a very good and well-illustrated description of what we may discover by inspection. Then come chapters on palpation, the study of the pulse, percussion, and auscultation, first as regards the organs of respiration and then of the heart. Part II. is concerned with the various diseases of the heart and their physical signs, while Part III. deals in the same way with the diseases of the lungs and pleural cavity. Dr. Cabot says that there is no other method of physical examination which needs so much practice as percussion, and he very truly adds, "none that is so seldom thoroughly learned." It is curious, indeed, to notice how seldom physicians attain such a mastery of the art as to feel confidence in the slighter indications given by this method unless confirmed in other ways. Some good and well-illustrated instructions are given as to the manner in which percussion is to be carried out, and here we may say that throughout the book, wherever the written word can be made clearer by the use of pictorial illustration, a wood-cut of some sort or another is inserted, which adds greatly to its value. The pictures of the student carefully listening with a "kinked" tube, or with one leg of the stethoscope accidentally detached, are worth a page of letterpress in fixing upon the student's mind the errors into which he may fall unless he pays attention to little details. Under the head of auscultation we notice that Dr. Cabot says that the chief point to be learned is to disregard various irrelevant sounds, and to concentrate attention upon those which are relevant. This is very true, and he properly urges the student to experiment upon the nature and extent of what he calls "skin rubs" by deliberately moving the chest piece of the stethoscope and so educating his ear to the various sounds which may be accidentally produced in this manner. The same with muscle sounds: unless one familiarises oneself with these sounds, it is impossible to properly eliminate them when they intrude among those for which we are listening. Dr. Cabot lays considerable stress upon the physiological modifications of the heart sounds which may be met with without any necessary association with morbid changes in the circulatory apparatus, and among them he shows in tabular form the extent to which accentuation of the basic second sounds vary with age. In youth it is the pulmonary sound that is accentuated, while as age increases the aortic sound becomes the most pronounced, so that in attaching any diagnostic value to this sign the age of the patient must be taken into careful consideration. The second and third sections of the book, in which the various diseases are discussed in the light of the physical sign by which they are accompanied, will doubtless be of great assistance to the student. Needless to say this work is intended as a guide and companion to, and not as a substitute for, practical work; no book, and no expenditure of midnight oil can take the place of practical experience. A book may, however, be of great help in giving an intelligible explanation of what is discovered, in teaching a careful student what to look for, and especially in showing what such physical signs as are met with tell us as to the presence or absence of disease. In all these ways this book will be found a most useful guide and companion.

Practical X-Ray Work. By Frank T. Addyman, B.Sc. Lond., F.I.C. With 52 illustrations and 12 full-page plates. (London: Scott, Greenwood and Co. 1901. Price 10s. 6d. net.)

Although in large towns it is probable that for some time to come X-ray work will mostly fall into the hands of specialists, to whom the elementary portions of this book will be of no particular interest, there must be many men who, from the fact that they are not within immediate reach of the expert, will find it absolutely necessary to be able to run an X-ray apparatus of their own. To such this manual may be strongly recommended. It is very clearly written, the explanations given of the phenomena, so far as they can be explained, are set forth in language which is not too exclusively technical, and we have no doubt that anyone who invests in the apparatus will soon be able under the guidance of this book to obtain good results. X-ray work, however, as the author points out, is essentially a thing to be picked up by experience. No book can take the place of practical work, but this well-illustrated handbook will undoubtedly give the beginner such a good general knowledge of what is before him as will enable him to obtain that familiarity with the machine which is necessary before reliable skiagrams can bel produced. It is a capital introduction to an art which is becoming almost essential in surgical work.

An Atlas of the Anatomy and Physiology of the Child, with Explanatory Text. By D'Arcy Power, M.B.Oxon., F.R.C.S. (London: Baillière, Tindall and Cox. Price 3s. net.)

In certain languages "The Child" is placed in the neuter gender—an admirable arrangement—and Mr. D'Arcy Power being desirous, we may presume, not to shock the delicacy of his readers, has preferred, instead of describing the anatomy of either man or woman, to give that of the child, *i.e.*, the neuter human. For the teaching of anatomy and physiology in schools this gets over many difficulties, and we have no doubt that this Atlas will make itself very popular for this purpose. The Atlas consists of a series of superposed coloured plates, by the successive turning up of which one exposes layer after layer of the human frame, coming down at last to the ribs on the other side. It is very well done and the description pages are both interesting and trustworthy, a combination of qualities not always to be met with.

